# A framework for modelling and designing transparency systems: A case of a Vietnamese pork supply chain

**DOI:** 10.1016/j.heliyon.2023.e21095

**Published:** 2023-10-17

**Authors:** Ayalew Kassahun, Cor Verdouw, Jeroen Roomer

**Affiliations:** a Information Technology Group, Wageningen University & Research, Wageningen, the Netherlands; b De Heus Animal Nutrition B.V., Ede, the Netherlands

**Keywords:** Meat value chains, Tracking and tracing, Design framework, Supply chain orchestration, Traceability systems

## Abstract

The three major meat supply chains in emerging markets are traditional wet markets, integrated supply chains, and the more recent collaborative supply chains. Customers in these markets are increasingly demanding safe and high-quality meat, which requires more transparency in the supply chain. This paper presents a generic framework for modelling and designing transparency systems in meat supply chains, with special attention to the needs of emerging markets like Vietnam where all the three supply chain types co-exist. The framework consists of *domain*, *product flow*, *business control*, *business process* and *transparency data* models. The main novelty of the proposed framework is its complementarity to cross-industry reference architectures and generic traceability standards, and its stakeholder-centric approach. The framework is demonstrated in the three pork supply chain types that are also widely present in Vietnam and are representative of the pork supply chains of emerging markets in general. The applicability of the framework is described in detail in a case study of a collaborative supply chain of independent members, which is one of the three pork supply chain types. The case study is selected for detailed analysis because the members work closely together to provide safe and traceable pork meat to consumers.

## 1 Introduction

In mature markets such as Europe and the US, meat products are relatively safe due to high food safety standards that are checked by advanced quality control systems. As a result, consumers in these countries can generally trust their meat products. However, the same cannot be said about meat products in emerging markets. The World Health Organization (WHO) estimates that about 600 million, almost 10 % of the world population, fall ill after eating contaminated food of which about 420 thousand people die every year [1]. The highest burden of foodborne disease was observed in Sub-Saharan Africa and Southeast Asia [2].

Meat supply chains in Southeast Asia, which are the focus area of this research, are vulnerable to disease outbreaks among consumers [[3], [4], [5]]. Disease outbreaks among animals, as is recently the case with the spread of African swine fever [6], may pose no threat to human health but cause major disruption to the supply chain and consumer confidence. In these emerging markets, the growing societal requirements for higher quality meat contrast with the recurring safety issues. As their income grows, consumers in emerging markets can increasingly afford high-quality meat and tend to move away from meat products of unknown origin sold at wet markets [7,8]. Consequently, there is a higher need for more transparency about the safety and origin of meat products they consume. In order to retain consumer trust, meat processors and retailers need to know in more detail what processing steps were executed, what ingredients or resources are used in these steps and how quality and safety are guaranteed in the processing of the meat products they trade.

A major reason for meat safety concerns in Southeast Asia is not only a lack of proper safety systems but also a lack of accurate meat safety information [9,10]. Most of the transparency literature on meat is on advanced traceability systems in developed countries. However, these systems cannot simply be copied to emerging markets. Supply chains differ substantially, for example, due to technical limitations, fragmented regulations, limited availability of processing facilities and lengthy supply chains including many small intermediate stakeholders [11,12]. Traceability in these types of supply chains is relatively under-researched. To gain better insight into the specifics of meat safety and transparency in emerging countries, this research conducted a case study of a pork supply chain in Vietnam.

Southeast Asian countries are the largest producers and consumers of pork meat [13]. As in most Southeast Asia, the Vietnamese meat market is mainly characterized by a large number of small-scale actors including backyard farms and small slaughterhouses [14]. However, the demand for high quality meat has been increasing [15,16]. Consumer demand for higher quality and safety of meat can only be addressed when there is transparency across the supply chain [17,18].

There have been several initiatives to implement information systems in the meat supply chains [[19], [20], [21]]. As the case study in this research indicates, markets that are largely controlled by a dominant actor (which has also an integrator or orchestrator role) offer transparency solutions in emerging markets. However, with the increasing maturity of the supply chains, the specialisation of the actors, and the wish of the actors to have an alternative to the integrators, decentralized and collaborative supply chains are arising, which are the main topic of this research.

It is our observation that the initiative to adopt a transparency system in Vietnam is driven by major value chain stakeholders, such as feed suppliers, large farms, and meat processors, who often have the capacity to gather tracking-and-tracing data at their facilities and from their supply chain partners. Some of these actors are providing Farm Management Information Systems (FMISs) to their contracted farmers in order to have better visibility and quality control in their supply chain. Likewise, slaughterhouses are increasingly deploying modern slaughterhouse information systems and industrial meat processors and retailers often have advanced information systems in place.

While transparency systems are being deployed across the supply chain, the missing link to realizing chain-wide transparency is the lack of standardisation and the inability of the different systems to share data. Against this background, the aim of this study is to analyse the state of the pork supply chain in Vietnam, offer a framework for modelling and designing transparency systems, and demonstrate the framework by applying it in the design of a transparency system for a collaborative supply chain. Specifically, the research questions we address are.

RQ1 How can a framework for supply chain transparency be designed?

RQ2 What are the current transparency scenarios of the pork supply chain in Vietnam?

RQ3 How can the framework be applied to enhance transparency in the pork supply chain?

To address these research questions, we identified three types of pork supply chains in Vietnam, that vary from the traditional wet markets to modern collaborative supply chains, and modelled the supply chains in order to capture the design alternatives for transparency systems in emerging markets. The existing modelling approaches and abstractions were inadequate in modelling and representing chain-wide transparency in supply chains, and therefore, we adapted and extended existing modelling views. The views are demonstrated in detail in a case study of a collaborative supply chain. Our approach and results are described in the remainder as follows. Section 2 presents a short background about traceability systems and enabling technologies. In section 3 we describe our methodological approach. In section 4, we present the state of the pork supply chain in Vietnam. In section 5, we describe the collaborative supply chain in Vietnam, which represents the future trend. Finally, we present the discussion of the results in section 6 and concluding remarks in section 7.

## 2 Background

### 2.1 Traceability in meat supply chains

Transparency and traceability are crucial for the functioning of meat supply chains. Transparency is a broader term and can be defined as “… the extent to which all its stakeholders have a shared understanding of, and access to, the product-related information that they request, without loss, noise, delay and distortion” [22]. Traceability is a more specific concept and refers to the ability to trace a product or a batch where it, its parts and ingredients have come from across upstream the supply chain and where it is now or in which products or batches it has currently been processing in Refs. [23,24].

There are many sector-specific characteristics that complicate tracking and tracing, such as the following, which also apply for the meat sector [22,25].

• High flow complexities characterise the food sector, as food supply chains consist of continuous product flows (bulk products traced in volumes or masses) and discrete product flows (packages traced in units). Traceability systems have to combine bulk products upstream of the supply chains with packaged products downstream of the supply chains.
• Batches and lots of food products have to be processed separated to prevent cross-contamination, which is not easy for continuous flows in food supply chains.
• Diverging and converging processes and flows characterise many food supply chains. Often food products are composed of many ingredients (including packaging) that all have to be tracked and traced, including many waste- and by-products.
• Food supply chains are often highly connected to each other, as waste and by-products of one supply chain are sometimes used as inputs in other supply chains.
• Food products are often highly perishable, especially fresh foods. Consumers and society impose stringent requirements on the quality and safety of food through food safety regulations and quality standards. Traceability data must therefore also refer to best-before-dates, country of origin of all ingredients, and certification of quality, veterinary, phytosanitary and ecological checks.
• Food supply chains are complex networks consisting of small and medium size enterprises (*i.e.*, farms, processing companies and retailers) that interact with multinational companies providing input to the supply chain and doing its retail. To ensure year-round supply of products, supply chains are often international, which makes tracking and tracing even more complex.

### 2.2 Traceability systems

Global Standard One (GS1), a global supply chain standard setting organisation with over two million businesses as members, identifies three sets of technologies for realizing traceability systems: object identification, data capture, and data sharing technologies [26]. Object identification technologies typically include barcodes including databar, QR code and data matrix systems [27]. Increasingly RFID (Radio Frequency Identification) tags [28] are being used in the pork sector. Object identification technologies use either labels attached to objects or increasingly a unique identifying characteristic, or biometrics, of the object, such as face, DNA, body shape, voice, movement, or the combination of any of these.

Data capture refers to the registration (or capturing) of data about the object using the object identification mechanism. Data capture has two main characteristics. First, besides the object ID, data capture registers other relevant data, some of which are necessary, such as the datetime and place when the data is captured, and others are optional but useful, such as why data is captured, what happened to the object, etc. EPCIS (Electronic Product Code, EPC, Information Services) is a global standard of GS1 for creating and sharing visibility event data, both within and across enterprises, to enable users to gain a shared view of physical or digital objects. Events contain data about the identity of the product, the date and time of event occurrence, the location where it occurred, and the reason why the event occurred. These are commonly known as the *what*, *when*, *where,* and *why* of transparency data. Second, data capture is ideally done automatically, and the use of manual data entry must be minimized. Tag readers are being used throughout the logistics processes and increasingly sensors are being used to capture many optional but useful data items [29].

Data sharing is a key element of traceability and requires not only the actual sharing of data (either by providing access to own data repository or sending the data), but, and most importantly, agreeing on a common standard for data and the interfaces through which data is shared. The GS1 set of standards and particularly the EPCIS standard are key requirements for data sharing [29].

There are three traceability approaches that differ structurally based on the enabling technologies and standards they adopt for supply chain traceability [30]. The first approach aligns with the basic EU requirement that each food supply chain partner should be able to trace back the origin of the input products it processed and the destination of its output products (commonly called the *one-step-back, one-step-forward* principle). According to this principle, data on food items and associated processes follow the same paths as the food products. Every supply chain actor is responsible for correctly passing the data it received and the additional information it generated to the next actor. Currently, this happens to a large extent paper-based using delivery notes. Every actor has access to traceability data about their customers and suppliers in their own internal systems [24].

The second approach is based on a shared repository of traceability events. The shared repository captures all events of processing and movement of food items across the supply chain, store the events on one or more EPCIS repositories, and make them accessible on a portal with the help of software tools for querying, discovering, and aggregating data. Different types of tools cater to the needs of different types of stakeholders, such as food authorities, supply chain partners, and consumers. Supply chain partners are usually willing to share data only with those parties that they are directly dealing with. For this reason, data governance agreements are established and authorization, integrity, and security mechanisms are implemented [31].

The third approach involves a distributed ledger in which traceability data are managed collaboratively by multiple participants. In this approach, the blockchain technology enables multiple participants to have an identical copy of the data and as a consequence, there is no need for a centralized database. Validation of the transactions is performed by the network based on a tamper-proof consensus mechanism, and not through a central intermediary. In a blockchain each transaction is encrypted and cannot be modified after approval, making this solution secure and tamper-proof. Due to these qualities of the blockchain technology, several studies have explored the transparency and security that the technology offers for ensuring food safety, integrity, and quality [32].

### 2.3 Modelling traceability systems

Meat traceability systems have to be able to deal with a high supply chain complexity. In order to get a grip on this complexity, it is essential to start the development of meat traceability systems with a systematic modelling of meat supply chains. Modelling frameworks are valuable means to do this in a precise and unambiguous manner. They define a coherent set of architectural views with the primary objective of creating a common understanding of the system among all stakeholders and guiding the development of the system [33,34].

A few modelling frameworks have been suggested to model supply chains. Probably, the most widely recognized and used framework is the SCOR (Supply Chain Operations Reference) model [35]. The SCOR model is focussed on business processes which are the operational level representation of a business.

To establish a connection between business processes, management processes, and business strategies on one hand, and process control and data capture at the workplace on the other hand, a hierarchical view is essential. The process hierarchy view consists of the management information layer at the top, the operation execution layer in the middle and the process control layer at the bottom [36]. The operational execution layer can further be divided into two: the Process Flow Model (PFM) and the Business Process Model (BPM). PFM captures how the individual business processes are inter-related; BPM zooms in the business processes and shows how activities and events form a business process. In PFM, the concept of Decoupling Point (DP) can be used to distinguish demand-driven and planning-driven sections of the PFM [37].

Business process modelling is a widely used and practical approach for capturing the activities and events that lead to a particular service or product outcome. The outcomes captured by BPMs are often simple but tangible, for instance, product is ordered, or product is shipped from store. The Business Process Model and Notations (BPMN) is a mature standard and widely used method to model BPMs [38].

In this study we will use traceability standards and supply chain modelling approaches to offer a modelling framework for traceability systems. The framework will be applied in a Vietnamese pork supply chain case study.

## 3 Methodology

The research is based on design-oriented and case study methodologies. A design aims to build purposeful artefacts that address heretofore unaddressed or unsolved problems [39,40]. A design methodology will be applied to derive the framework. A case study will be used to demonstrate the applicability of the framework.

### 3.1 Framework design

The design artefact developed in this paper is a framework for modelling transparency systems for meat supply chains in emerging markets. Design-oriented research typically deals with the ‘how’ questions, *i.e.,* how to design, construct and apply a new design artefact for a class of problems [41,42]. We designed the framework following the ISO/IEC/IEEE architecture framework [34] for a stakeholder-centred modelling approach. Following this approach, which is widely used in the software industry, an architecture must address the concerns of a class of stakeholders whose viewpoints must be captured using models. The models developed for a class of stakeholders are called *views*.

In this study we adopt the five design criteria for designing a reference model for meat supply chains [36]. These are: (1) the model must be generic, *i.e.,* the model must be an abstraction and not a representation of a specific case, (2) the model must be modular, *i.e.,* it should be composed of smaller models that address specific needs of specific stakeholder type, (3) the model must be executable, *i.e.,* besides having a visual representation, the model should be executable by, or convertible to, a software tool, (4) the model must be configurable. *i.e.,* non-technical users should be able to tune the model to their needs by changing parameters, and (5) the model must be applicable in more than one application scenario or domain.

We present five hierarchical modelling views and generic models based on existing generic frameworks in order to fulfil the design criteria of genericity and modularity. The two major generic frameworks from which our modelling framework is derived are the SCOR model [35] for modelling supply chains and the GS1 transparency standards [26] that provide generic models for capturing and sharing transparency data. We derive our framework by combining and adapting modelling approaches from these major sets of standards.

Based on the framework modelling approach of Verdouw et al. (2010) we develop the modelling views that belong to 5 hierarchical levels, which are the *domain*, *product flow*, *business control*, *business process* and *data* views. The domain and product flow views capture the supply chain system. The business control view enables the modelling of various configurations. Both the business control and business process views provide fine-grained models that allow modularity in the software system that implements the framework.

### 3.2 Case study

A case study approach from a real-world application context [[43], [44], [45]] has been adopted in this research. Our case study involves three supply chain scenarios found in the Vietnamese pork sector: (1) traditional wet market supply chains that are generally open for all stakeholders to join, but uncontrolled in terms of quality and food safety, (2) integrated supply chains that are largely controlled by a central orchestrating company, and (3) emerging modern collaborative supply chains, that differ from integrated supply chains in the sense that each supply chain participant continues to operate as an independent partner in the value chain.

In Vietnam, but also in most emerging markets, the dominant sales channel for meat products is the wet market. The wet market supply chain is characterised by many small-scale actors, including small-scale feed mills, small-scale traditional farms (also known as backyard farms), dealers, small and unsophisticated slaughterhouses, and small retailers. The resulting fresh meat products are sold in traditional open-air markets where vendors also sell various other products in addition to meat. Next to the shopping function the wet markets are often considered as cultural and social hubs where people gather to socialise and share information.

In integrated supply chains, the central orchestrating companies control major aspects of the meat production supply chain, which include at least two of the following supply chain activities: breeding, fattening, feed production, slaughtering, meat processing and retailing. These integrators may also provide better pig breeds, technical support and credit lines to their contracted farmers. The emerging modern collaborative supply chains, which are the main focus of this case study, consist of specialized and decentralized meat product operators that are governed by a common set of quality standards.

The case study is conducted at a feed company that is active in a modern collaborative supply chain. The company operates many production locations in Vietnam and is one of the top three animal feed producers in the country and has in recent years expanded into other Southeast Asian countries. Due to the concerns over the safety of meat products in Asia, the company is collaborating with various stakeholders to realize modern and transparent collaborative supply chains. As part of this effort, the company has developed and deployed a Farm Management Information System (an FMIS) for its customers (which are farmers) and a Slaughterhouse Information System (an SIS) for use in collaboration with another food operator in the operation of slaughterhouses. We monitored the development of the FMIS and other transparency solutions, as well as the transition towards a collaborative supply chain at the case study company, both as researchers and as the responsible manager of the systems.

The analysis done in the case study is based on *facility visits*, *interviews*, and *modelling*. Four facilities, one of which is owned by the case company, were visited. The facilities were selected for visits because they were related to the case study company and the latest available information technologies were used in the facilities. The facilities represent the four key phases in meat supply chains, namely, feed production, breeding, fattening, and slaughtering. A chicken slaughterhouse was visited in order to gain additional insight into integrated supply chains in which the production of feed, breeding, fattening, and slaughtering is generally centrally planned and managed. The pig farm had, besides the breeding sows, also produces commercial hogs, works with contracted farmers, and operates own meat processing facility.

Before, during, and after the facility visits, a number of interviews were conducted with six experts of the case study company who had five different roles: FMIS specialist, strategic purchaser, project manager, software specialist, and sales support. The interviews were conducted to gain insights into the existing transparency systems in place and the plans for the future. Some of the interviewed persons have detailed knowledge about the current state of transparency and traceability in Vietnam, while others were involved in traceability innovation projects. Several interviews were conducted with all interviewees to make a better sketch of the current situation of transparency and traceability and gain insight into possible future scenarios.

We enhanced the modelling framework based on the insights and information gained through the case study. The results of facility visits and interviews were mainly used to model the current state and possible future scenarios based on the proposed framework. First, the main transparency scenarios of the case study were modelled. Two dominant supply chain types in Vietnam are the integrated supply chains and the traditional wet markets. They represent the existing (AS-IS) scenarios in our modelling. The emerging future scenarios consist of collaborative supply chains. They represent the desired (TO-BE) scenarios.

## 4 A modelling framework for traceability

This chapter presents the designed framework by introducing its views. Each of the following subsections will shortly introduce a view and discuss a simplified model. A more detailed definition of the elements and relations of the models is provided in Appendix A.

### 4.1 Domain model

**Fig. 1:**
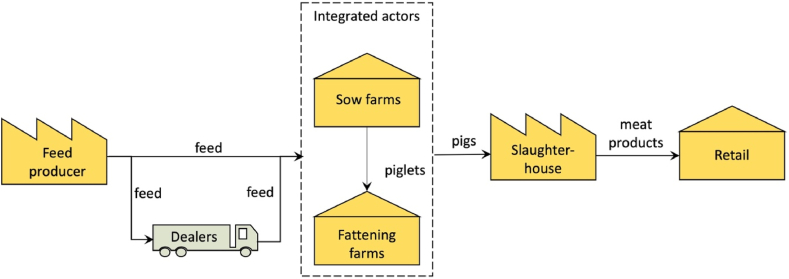
Domain view.

This view is meant for all stakeholders and serves to create the necessary initial common understanding among all stakeholders. A conceptual model is therefore a simple box-and-lines model where the meanings of the boxes and the lines are intuitive, and the model captures the essential elements of the supply chain. Fig. 1 shows an example of a simplified pork supply chain domain model, visualising the involved producers, farmers, intermediaries and retailers. Table A1 in Appendix A further defines the elements and relations of the domain model view.

**Table 1:** The main novelties of the proposed framework.

Related solution	Contribution	Main novelties of our framework
SCOR [35]	Cross-industry reference architecture for supply chain modelling	Adding a product flow view that allows modelling of traceability processes, traceable products, their flow in the supply chain, and their granularity.
GS1 traceability standards [26]	Widely accepted and adopted generic traceability models	Adding specific events and event data specifications tailored to food and meat supply chains.
ISO/IEC/IEEE architecture framework [34]	Stakeholder-centric modelling approach	Adding tailored views to model the architecture of supply chain traceability systems, which are domain, product flow, business control, business process and transparency data views.
Diverse supply chain traceability systems	Sector specific traceability systems and proof of concepts	The framework enables systems designers and researchers to understand and explain various supply chain transparency systems, as these systems can be represented by one or more views within this framework

### 4.2 Product flow model

**Fig. 2:**
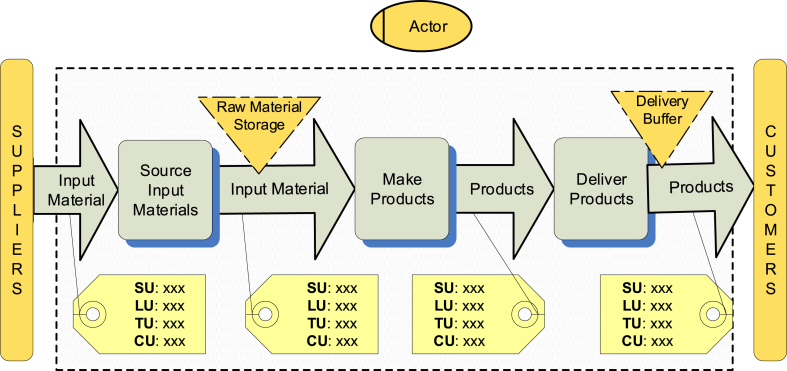
Product flow view.

The product flow view is adopted from the supply chain framework of Verdouw et al. (2010) and is meant for all stakeholders involved in the actual tracking and tracing of products. It visualises a value chain, *i.e.*, a system of interlinked business processes, each adding value to the product or service for the end customer [46]. In a supply chain, value-adding processes are performed by multiple firms, especially if there is a high degree of specialisation. Each value-adding process transforms inputs into outputs. An output can be another product, an adapted product (changed properties) or the same product in a new location or presented in a new form. As such a product flow model visualises the physical flow from input material to end products delivered to the final customer. Fig. 2 shows an overview of a generic example of one of the supply chain actors.

The value-added processes shown in Fig. 2 are defined based on SCOR level 1 process definitions. A product flow model must include at least one source process and one deliver process for each actor. The processes can be renamed in such a way that they are recognizable for users (*e.g.*, make = slaughtering). Producers also must include at least one make process. Every arrow represents a product or products (input or output). In the generic model presented in Fig. 2, there are only two types of products (input material and products), and in actual product flow actual product names can be used (*e.g.*, product = half carcass). Products can be stored in strategic stocks (depicted as upside-down triangles), and in this example, a storage of input material and a delivery buffer of end products are included. Table A2 in Appendix A further defines the elements and relations of the product flow view.

The product flows comprise different levels of granularity, and each can uniquely be identified by particular identification standards. In this model we adapt the traceability layers and related standards of GS1 [26].

1. Shipping Unit (SU): an item or group of items that are delivered together to a location. SUs can be identified with Global Shipment Identification Number (GSIN). In addition, shipments include shipping and receipt locations, that are identified with Global Location Number (GLN).
2. Logistics Unit (LU): mostly associated with a pallet represents an item composed of one or more products for the purpose of transport or storage. LUs are generally identified with Serial Shipping Container Codes (SSCC).
3. Trade Unit (TU): a product, a package of identical products, or a composition of products that are traded between businesses. TUs are typically identified with Global Trade Item Numbers (GTIN). If each item or a batch of items has to be uniquely identified, GTIN in combination with a serial number or batch/lot number identifies the item or the batch uniquely. The combination of a serial number with GTIN is referred to as Serialized GTIN (SGTIN).
4. Consumer Unit (CU): a product as it is sold to end customers. CUs are identified in the same as Tus.

### 4.3 Business control view

**Fig. 3:**
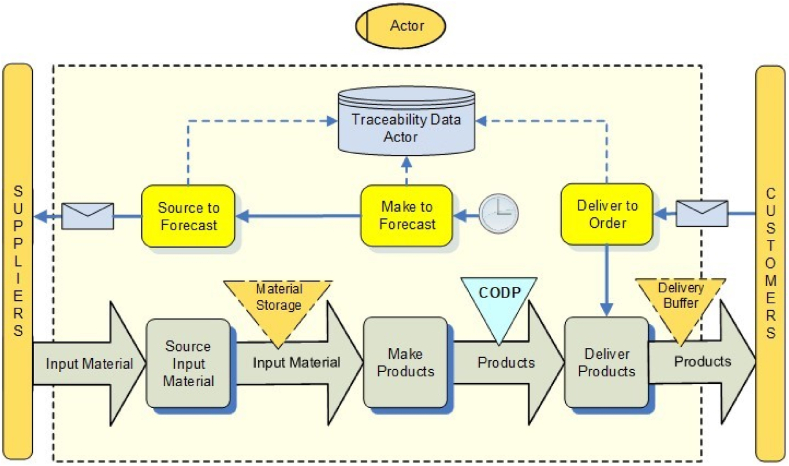
Business control view.

The business control view is based on thread diagrams in the framework of Verdouw et al. (2010) and provides a high-level process overview of the chosen supply chain configuration (Fig. 3). The business control view is used by those stakeholders who are involved in the redesign of the supply chain. In addition to product flow models, this view shows how the business processes are triggered and controlled, *i.e.*, how they are planned and managed in such a way that the business processes in a supply chain are controlled. A major control challenge is how to balance lean control that anticipates future demand and agile control that focusses on quick response to unpredictable demand [47,48]. A key factor for finding this balance is the Customer Order Decoupling Point (CODP), also called order penetration point [49,50]. The CODP separates that part of the supply chain geared towards directly satisfying customer orders from that part of the supply chain anticipating future demand [37].

The CODP position is also a key factor for traceability because it defines where the link between material flow and customer order information ends, *i.e.*, it defines the boundary between state-dependent and state-independent data [51]. Traceability of state-dependent data needs a unique identification, that differs per product or batch of products. For traceability of state-independent data, static identification is sufficient. For example, the product definition (main structure and features) for make-to-stock (MTS) situation is usually state-independent. Consequently, product types can be identified with a standard article code that does not change (*e.g.,* GTIN). A state-dependent identifier is needed to track and trace particular products in the supply chain (*e.g.,* SGTIN that adds a serial number). When moving towards more upstream CODP positions including make-to-order (MTO) product data become more dependent on customer orders.

Fig. 3 shows an overview in a generic example of a single supply chain actor. It starts with depicting the position of Customer Order Decoupling Points (CODPs) in a Product Flow Model. The CODP in this example is downstream of the Make process, which implies that this actor produces end products to stock, while the delivery is done to customer order. Next, the Control Business Processes (yellow rectangles) are added for each step of the value chain. A Control Business Process represents a sequenced group of business processes that follow the same control strategy. They are defined on SCOR level 2 and can be either responsive (triggered by an order, envelope symbol) or anticipatory (triggered by a demand forecast, timer symbol).

Besides, the traceability information dimension is added by visualising the source and destination of direct and indirect traceability information in data flows (dashed lines) and where traceability information is stored, either within a particular company or on a supply chain level (database symbols). Table A3 in Appendix A further defines the elements and relations of the business control view.

### 4.4 Business process view

The business process view depicts the details of the business processes identified in the business control view. It shows the sequence of activities and the flow of information along the flow of activities. The business process view is based on the Business Process Modelling Notation (BPMN) and this choice ensures a smooth translation of supply chain designs to information systems architecture (OMG, 2009). BPMN aims to provide a standard notation readily understandable by all business stakeholders, including business analysts who create and refine the processes, the technical developers responsible for implementing them and business managers who monitor and manage them. BPMN also provides a mechanism to generate an executable business process using Business Process Execution Language (BPEL) from the BPMN models. BPMN's four basic element categories are.

• *flow elements*: events (start, intermediate and end), activities, and gateways;
• *connecting elements* (arrows): sequence flows, message flows, and associations;
• *swim lanes*: pools and lanes; and
• *data elements*: data objects (input and output), data stores and messages.

**Fig. 4:**
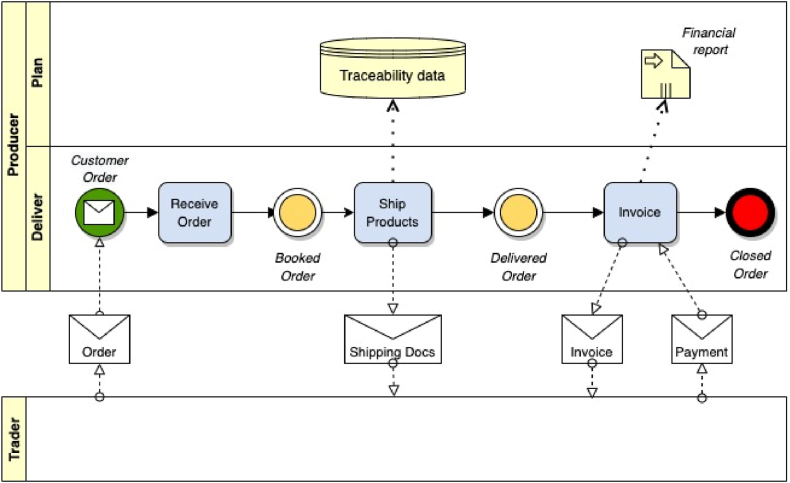
Business process view.

Fig. 4 visualises how these elements are applied in a generic example of a business process diagram.

This diagram zooms in on the ‘Deliver to Order’ business control model in Fig. 3 in order to illustrate how the different models are interlinked. It shows the sequence of activities from start (customer order) to the end (closed order), which always should be logical, clear and self-explaining. In the activity ‘Ship Products’ the shipped items are scanned, which results in an entry of traceability data.

In the latest version of BPMN (2.0) many specific variants of the elements are defined, mainly for technical reasons. In order to keep the models understandable for also business users, the method proposed in this paper uses only the basic BPMN elements, as defined in Table A4 of Appendix A.

### 4.5 Data view

**Fig. 5:**
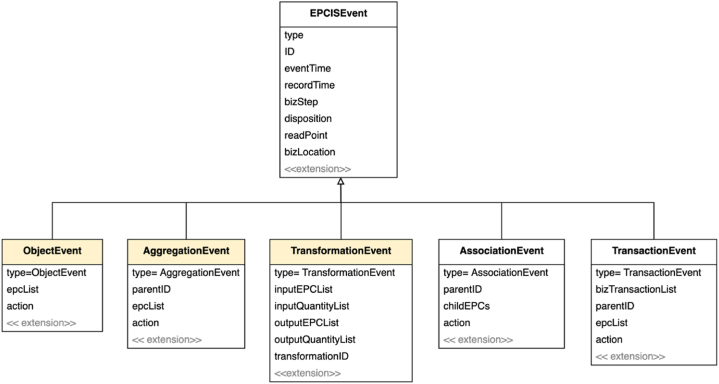
Transparency data model.

The data view is used to identify the data entities that constitute traceability information. The data model used in the framework is based on the EPCIS event model of GS1 and its extension developed at IoF2020 meat transparency and traceability use case [52,53] and represented using a UML class diagram [54] as shown in Fig. 5. The meaning and significance of the data model in the pork supply chain are described below.

The EPCIS standard defines five specialized event classes, namely: object, aggregation, transformation, association, and transaction events. The five event types have many common attributes which are depicted in the figure as a super class EPCISEvent. Common attributes across all event types are the *ID* (the event ID that uniquely identifies the record of the event), *eventTime* and *recordTime* (the times the event occurred and when it was actually recorded), *bisStep* and *disposition* (representing why the event occurred, the first identifying the business process and the second describing what the status of the object is after the event), *readPoint* and *bizLocation* (representing the place of the event, the first representing where data about the object is read and the second representing the location identification of the event).

The *ObjectEvent* represents the observation of an object or objects and is by far the most common event. When pigs are purchased, piglets are weaned, and when they get ill, treated, loaded on a truck for transport, *etc.*, the corresponding data are registered as object events. The second type of event that often occurs in the meat sector is the *TransformationEvent*, such as slaughtering or cutting. This event represents the transformation of one or more inputs into an entirely new product or products. The use of transformation event in practice is limited because it is a new addition to the standard and users have already workarounds in place that capture facts about transformation using object events. A common event during transport is the *AggregationEvent*, in which one or more objects are associated with the container (*e.g*., packaging). Somewhere in between the aggregation and transformation event is the *AssociationEvent* with which a strong physical association (or disassociation) of objects (*e.g.*, organs) with the parent object can be captured. Last, a *TransactionEvent* enables to capture the association (or disassociation) of objects to the business transaction and can be used to link the physical events to the management processes of business control (see section 4.3). Table A5 of Appendix A further defines the used elements and relations of the data view. The use of generic event models provides a mechanism to extent domain specific events and use them without the any further modification to the underlying transparency system providing extra mechanism for configuration.

## 5 Case study: application of the framework

This section describes how the framework is applied in the case study involving the three major pork supply chain scenarios that are used in Vietnam. We first apply our framework to the wet markets and an integrated supply chain briefly in section 5.1. and present a detailed design of the collaborative supply chain markets in section 5.2. The collaborative supply chain was chosen for a detailed analysis since it represents a desired future scenario where transparency and traceability will be enhanced.

### 5.1 Modelling the current state

#### 5.1.1 Transparency in wet markets

The wet market supply chain refers to the pork supply chain where the final fresh meat product is sold on open-air markets with different vendors selling a diversity of food items. Currently, many consumers in Vietnam prefer buying fresh meat from the wet market and many of them have built enduring relationships with their fresh meat suppliers in these local wet markets.

Wet markets are often supplied by smallholder farms, many traders, and unsophisticated slaughterhouses. The smallholder farmers are often family run and operate on a limited scale, therefore, producing a limited number of pigs. These farmers generally purchase feed from dealers and occasionally directly from feed factories. The dealers buy feed from feed companies and retail it to farmers. Besides their role as logistics providers, dealers often provide financing to the smallholder farmers. The fattening farms raise piglets, that are sourced from breeders, who keep a large number of sows to breed piglets for sale. Fattened pigs are commonly sold to traders who sell the animals either to small (backyard) slaughterhouses, large professional slaughterhouses or directly to wet markets. When pigs are sold to small slaughterhouses there are only limited quality requirements, while large-scale professional slaughterhouses often have higher quality requirements.

**Fig. 6:**
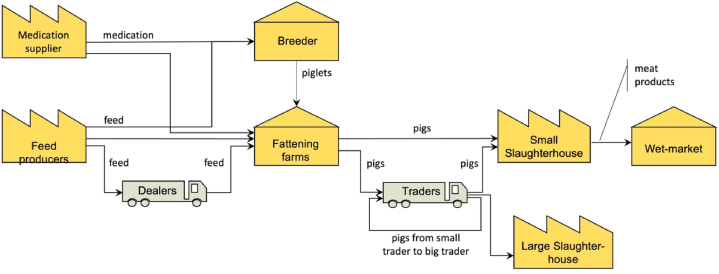
Conceptual model of the Vietnamese wet market pork supply chain.

Small-scale traders also sell pigs onwards to larger-scale traders. Large-scale traders transport the pigs to large slaughterhouses, or they export the pigs. Meat from small slaughterhouses is supplied to the wet markets. While feed dealers play the role of retailer, pig traders play the opposite role, as aggregators. The other important actors in the wet market (and the other supply chain scenarios) are medication suppliers. In the wet market, there is little data and transparency in the use of medications, and the little information there is, it gets lost because dealers and traders generally track their transactions insufficiently and therewith form the weakest link in the supply chain. The domain model of the wet markets is depicted in Fig. 6.

The domain model shows that wet markets lack a supply chain coordinator or orchestrator, which is a defining characteristic of the integrated and collaborative markets. Furthermore, there are relatively many intermediary actors that trade and ship products between producers. Traceability is hardly possible in this scenario. Record keeping at feed dealers, pig traders, and retailers are virtually non-existent. If a traceability system is existing, it is generally weak. Generally, a record of the chain of custody is lost when dealers and traders are involved. Dealers and traders generally receive cash payments and may not keep the record of buyers (in the case of feed dealers) and sellers (in the case of pig traders).

#### 5.1.2 Integrated supply chains

**Fig. 7:**
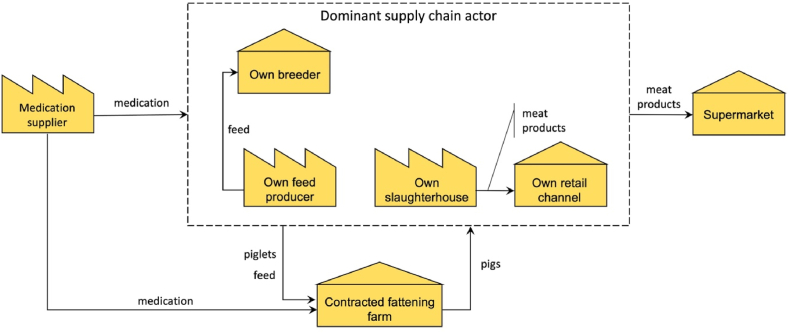
Domain model of the Vietnamese integrated pork supply chain.

In an integrated supply chain, one dominant actor performs, or has a significant influence on, a major part of the production activities of the supply chain. Fig. 7 depicts a conceptual model of this supply chain scenario. Various integrated actors cover different sections of the pork supply chain, and the figure covers the common activities covered by most integrated actors. For instance, a meat processing company may also own breeding and fattening farms. The case study company, for instance, is a feed producer and supplier with partner breeding farms and meat processing facility.

The characteristic feature of the product flow in the integrated market is that the dominant (the integrator) actor controls the production at key stages of the supply chain and gains significant insight into the chain of custody of products. A dominant actor that contracts out the fattening process to smallholder farmers play a major role in quality control. It achieves this, for instance, through its farm coaches, who guide farmers on how to implement best practices, and through an FMIS it supplies to farmers, which captures data at from the farmers and stores it centrally at its information system. The dominant actor may in some cases sell part of the meat products through its own retail channels.

In the integrated supply chains, transparency is achieved in almost all cases with the help of a transaction system the dominant actor uses. The transaction system in use is generally a propriety information system, such as an ERP (Enterprise Resources Planning) system, in which the data is not necessarily captured in compliance with any transparency standards described in sections 2.2, 4.2, 4.5. The dominant actor can in many cases track meat products in the retail shop to a specific farm or even animal, as in the case in the case study company. Depending on the needs and the influence of the dominant actor, meat products can even be traced back to the specific breed (sow and boar), feed consumed, and medication used, if any.

### 5.2 Modelling a collaborative pork supply chain

The rising health-conscious middle class in Vietnam is increasingly willing to pay more for safe high-quality meat products, and tends to shop in supermarkets instead of wet markets [55,56]. This has opened new possibilities for the professionalisation and specialisation of meat product operators and the rise of complex and collaborative pork supply chains. To accommodate this rising demand, the case study company has initiated different cooperation models including a joint venture with the aim to provide to consumers safe and traceable pork meat that is produced in a sustainable way. This initiative is presented in this section as an example of a collaborative supply chain.

#### 5.2.1 Domain model of collaborative supply chains

**Fig. 8:**
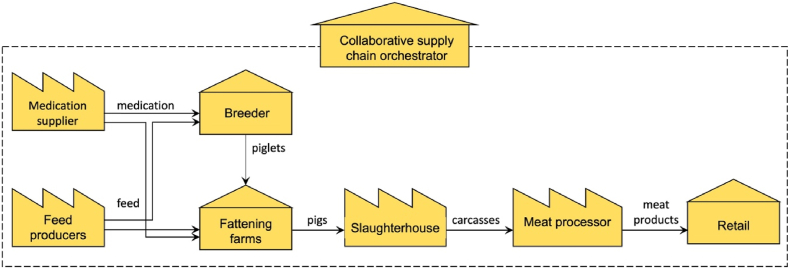
Domain model of a Vietnamese collaborative supply chain.

A simplified version of the collaborative pork supply chain of the case study is presented in the domain model depicted in Fig. 8. This scenario includes an orchestrator, which is an association that consist of representatives from the different independent supply chain members. This organisation is engaged in activities such as running a demo pig farm and demonstrating compliance to standards such as GLOBALGAP, a private standard developed and controlled by the orchestrator, VietGAP (an animal welfare standard in Vietnam) and Ethical Welfare Initiative (ETI). Actors should meet these standards in order to participate in the initiative. One of the roles of the orchestrator is thus developing and maintaining quality standards and performing regular audits to ensure compliance. Key to its operation is the sharing of transparency data among the independent actors following a common data sharing standard.

#### 5.2.2 Product flow model of collaborative supply chains

**Fig. 9:**
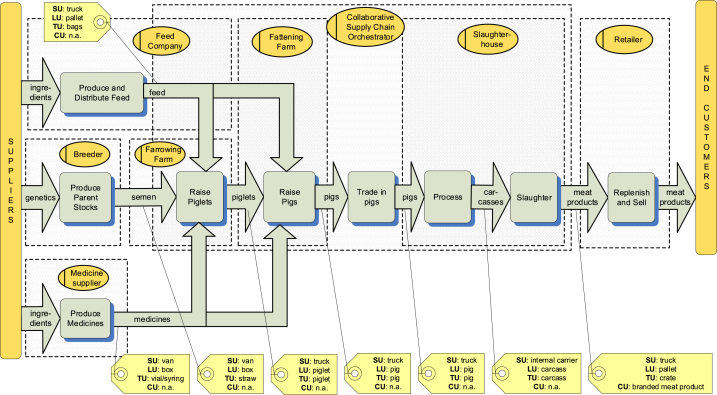
Product flow model for a collaborative pork chain in Vietnam, including examples of traceability units for the orchestrating company.

The product flow model for the collaborative supply chain of the case study is shown in Fig. 9. Nine value adding processes involving seven chain actors are included in the model. The suppliers beyond those shown in the figure, such as suppliers of ingredients for feed production, medication and genetic material for parent stock producers are considered out of scope and are represented by the vertical bar labelled *suppliers*. Likewise, the *end consumers* are represented undifferentiated by the vertical bar labelled *end customers*, but they could be consumers at home, at restaurants, or other places of meat product consumption.

The traceability units are presented only for the orchestrator's processes and describe two aspects of traceability: 1) internal traceability showing how input products to a meat product operator are linked to the output products of the same operator, and 2) external traceability showing how the output products of one set of meat product operators (suppliers) are linked to the input products of another set of operators (customers). For example, the FMIS system provided by the case study company to the farmers helps the farmers to track products (feed, animals) within their farms. External transparency is realized when data from the FMISs is shared to the SISs of the slaughterhouses, and vice versa, so that the identities of pigs at farms are linked to the identities of pigs received at slaughterhouses. The value-adding processes (depicted by rounded rectangles), and product flows (arrows) represent the two aspects of the transparency.

The traceability units are as follows. The breeders (farmers producing piglets) keep a record of nearly all events that are related to the cycle of individual sows, including new arrival and removal, mating, farrowing, weaning, fostering (adoption) and the registration of medication. At each of these activities, the tag number that uniquely identifies the animal is registered. By arrival, the birth date, the arrival date, breed, location and the company name of the supplier are registered, which enables the tracing of the animal to the supplier. By removal, the removal date and the reason are registered. When documenting mating, the tag numbers of both the boar and sow are registered. When farrowing, the sow tag number and the date, along with the number of piglets born in 5 categories (born alive, born dead, mummified, light weight and defect) are registered; the piglets are not individually identified at this stage. When weaning and fostering, the total number of piglets and their average weight are registered along with the tag number of the sow.

The next major process is *raising slaughter pigs* (or fattening). In the desired situation, the delivery of feed and the associated feed information is registered in the FMIS of the farm at delivery; and the same must happen for medication. Currently, the plan is to identify pigs per batch as the cost of registering and managing pigs individually using ear tags is prohibitive in emerging markets. To realize better traceability, the batches of pigs will not mix and both feed and medication usage are registered per batch of pigs. Once the pigs are fattened, they are sold directly to the slaughterhouse and mixing of pigs of different farms will not be allowed. However, external transport companies are used to transport the pigs from farms to slaughterhouses and the possible mixing of the animals during transport remains a challenge.

The next step of *trading in pigs*, executed by the orchestrator, will guarantee the information supplied by the farms are correct and are correctly delivered to slaughterhouses. At the slaughterhouse, detailed information about the individual pig and carcass is gathered and added to the data warehouse information system managed by the orchestrator. The slaughterhouse sells the products to wholesalers and supermarkets, and the labelling of the products ensures the individual product at the supermarket can be traced back to the batch of animals supplied by farmers.

#### 5.2.3 Business control model of collaborative supply chains

**Fig. 10:**
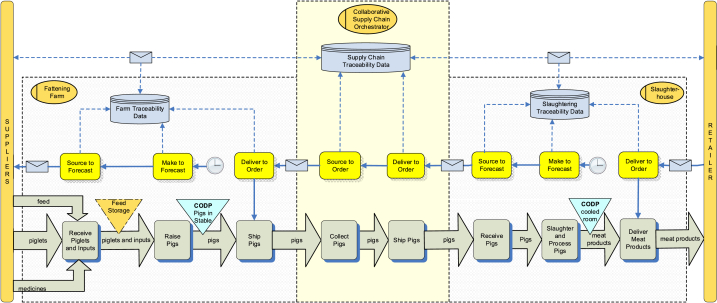
Business control model of a collaborative Vietnamese pork chain, an example of a fattening farm and a slaughterhouse that collaborate in supplying meat products to a retailer.

In the collaborative supply chain of the case study, an independent orchestrator has a key role in the coordination of demand fulfilment. Demand information is shared for the alignment of planning, control and operations of the involved actors. Fig. 10 shows a (simplified) example of a fattening farm and a slaughterhouse that collaborate in supplying meat products to a retailer. In this example, the slaughterhouse produces meat products to stock based on a demand forecast. The produced meat products are stored in a cooled room until the retailer order is received. The meat products are then picked and shipped to order. As a consequence, the first CODP in this scenario is the stock of chilled meat products in between the slaughtering and delivery process. The slaughterhouse orders the needed pigs from the orchestrator. The orchestrator collects all orders from slaughterhouses and allocates them to fattening farmers. They select the pigs that will meet the customer's quality specifications and ship them to the collection point of the orchestrator, which takes care of the delivery to the slaughterhouses. So, this implies that the second CODP of this scenario is in between raising and delivering pigs, *i.e*., the inventory of slaughter-ready pigs in the farm stables. The farm produces the pigs based on its own estimations, which are based on forecast data of the association as well as supply-related data, including stable capacity. All physical steps (receipts, transfers, production, shipments, etc.) in this process are logged for tracking and tracing purposes. The traceability data are shared with a supply chain traceability system of the orchestrating company. Authorized users of the system can monitor the status and take action when problems arise.

#### 5.2.4 Business process models of collaborative supply chains

In this section, business processes at a farm and slaughterhouse are selected for modelling and analysis. Both business processes are complex and thus a significantly simplified versions of the processes are modelled. The first business process modelled is a long-lasting fattening business process. The second business process modelled is the intake at slaughterhouse, and the third is the slaughtering business process.

**Fig. 11:**
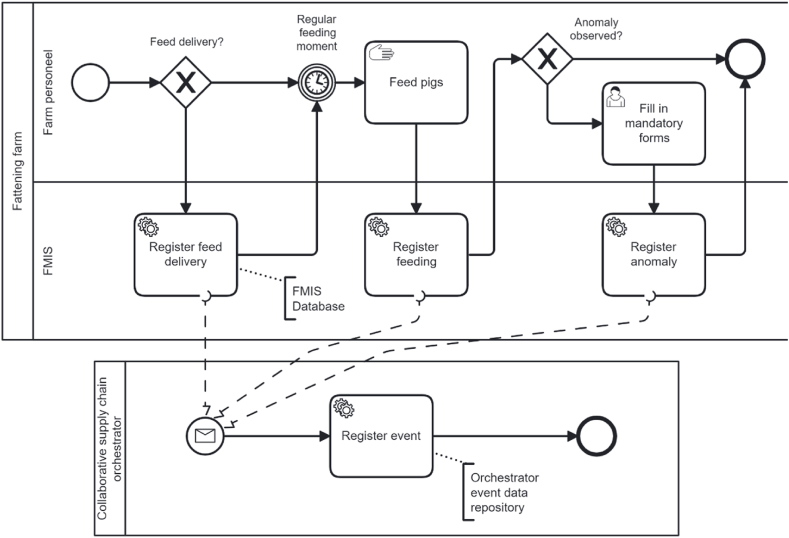
A business process model of registering events at a farm.

Fig. 11 shows some of the activities of the business process of registering routine feeding and observation activities at farm. At the case study company, the farmer places an order for feed supply when the farm runs low on feed. The order is registered in the feed factory's order management ERP system and the farmer's credit is checked. If the farmer has sufficient credit, the order is confirmed, and the feed can be picked up together with an order receipt; otherwise, the order is declined and the feed buying process is terminated. The order receipt contains the product codes (through which the details of the feed products can be looked up), details of quantities and unit prices, and other meta information such as date, credit term, method of payment, details of the feed company, and details of the customer. After picking up or receiving the feed, the farmer stores the feed and fills in the feed data in FMIS manually. At the time of the case study, there was no integration of the farmers' FMIS system and the feed supplier's ERP system. The farm employees feed pigs and though routine feeding activities are not registered in the FMIS, the system enables the registration of the feeding activities, including the feed type, the barn where the feeding took place, the time, the employee, and optionally the weight and other descriptive information. The anomaly observed is a collective name such as the registration of sickness and the supply of medication. The FMIS used in the case study is configured to transfer standardized transparency event data to the orchestrating company.

**Fig. 12:**
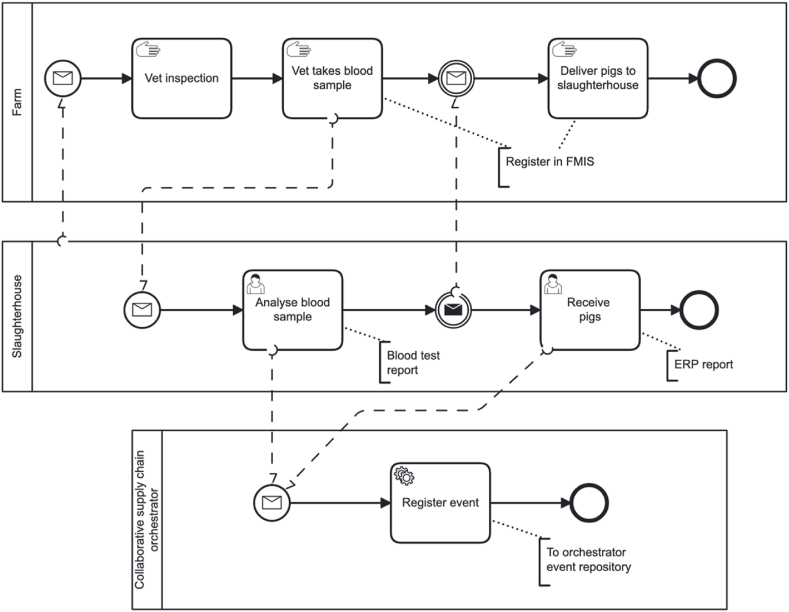
A business process model for the intake of pigs at a slaughterhouse.

The business process showing the delivery of pigs from farms and the intake of pigs at a slaughterhouse is depicted in Fig. 12. The model does not include the initial details for brevity. Before pig intake, the farmer and the slaughterhouse agree on the number and the weight category of the pigs, and the delivery time. The accountant of the orchestrating company creates a purchase order (PO) in the ERP system—these and other necessary steps are not depicted in the model to keep the model small and readable. The slaughterhouse sends a veterinarian to the farm to take a blood sample and check if the pigs are healthy. The veterinarian checks the pigs at the farm and sets up a veterinarian certificate. The farmer registers the certificate number in the FMIS. The blood is tested among others on the African Swine Fever (ASF) virus. If the blood sample passes the test, the pigs can be picked up at the farm. The slaughterhouse sends a truck to the farm. At the farm, the pigs to be delivered are weighed, counted, and loaded into the truck. The farmer creates a Sales Order (SO) in the FMIS, and the veterinarian certificate is attached to it. The data about the pigs and together with the veterinary certificate and the results of the blood test are all printed on paper and given to the driver. When the sales and purchase administrative steps are completed, the pigs are loaded on the truck.

**Fig. 13:**
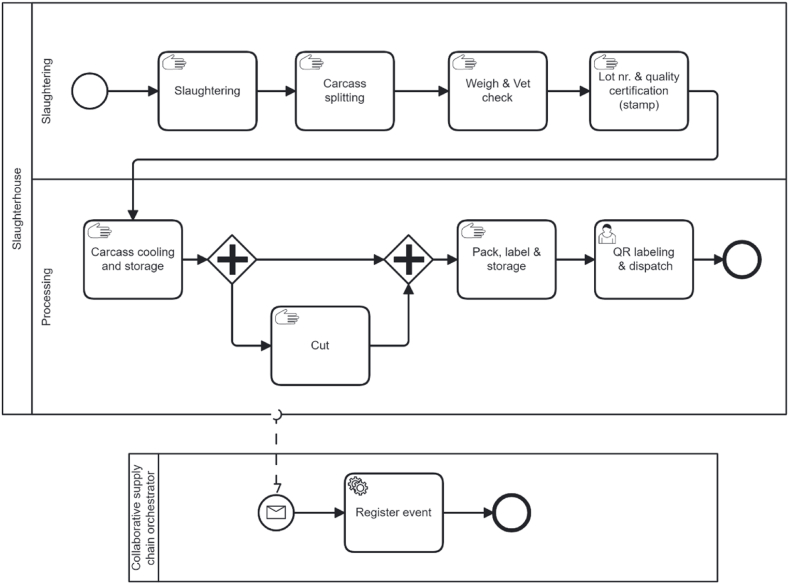
A business process model for activities at a slaughterhouse.

The activities at the slaughterhouse are shown in the business process model depicted in Fig. 13. Once the truck with pigs arrives at the slaughterhouse, the number of the veterinary certificate will be checked with the number that is recorded in the PO at the farm in the shared database of the orchestrator. If the number is correct, the truck can enter the slaughterhouse, otherwise it is rejected. On the terrain of the slaughterhouse the truck with the pigs is weighed. Then the truck drives to the penning area and the first veterinary check will be conducted during unloading into the penning area. The pigs are checked individually and are sorted into categories based on their conditions. If there are any dead pigs, they stay in the truck and the truck is weighed again with only the dead pigs. The dead pigs are dispatched to a special warehouse. Afterwards the truck is weighed empty. All weigh records are filled in the ERP system of the slaughterhouse. When all the data is filled in, a PO receipt is created.

In one pen, only pigs from the same truck are placed. It is possible that the content of one truck occupies more than one pen. All the pigs in one pen (or in one truck) are considered one batch. After the penning, where the pigs stay for at least 10–12 h with only water, the pigs are slaughtered. In the slaughtering process consists of several steps such as stunning, killing and cleaning, which are not included. The slaughtering process results in whole carcasses. After cleaning the carcass, products that are distinguished include the head, the blood, organs, and the carcass. The blood, which is treated as waste, and the processing of other by-products are not included in the model. The carcass is split up into two half carcasses and the half carcasses are weighed as a pair.

A veterinarian checks if the quality of the carcasses is good, and if not the entire carcass or part of it can be rejected. The carcasses recognized as good quality receive a stamp with the batch number that serves as a quality certificate. The heads and organs are put in plastic bags or boxes with a label with a barcode to identify the batch number. Before dispatch, the products are provided with a QR code. An external order release is created when a product is sold. The QR code contains information that is stored in the orchestrator's database. When the QR code is scanned, information about the slaughterhouse, farm of origin, production date, and the expiration date is provided.

#### 5.2.5 Data model of collaborative supply chains

**Fig. 14:**
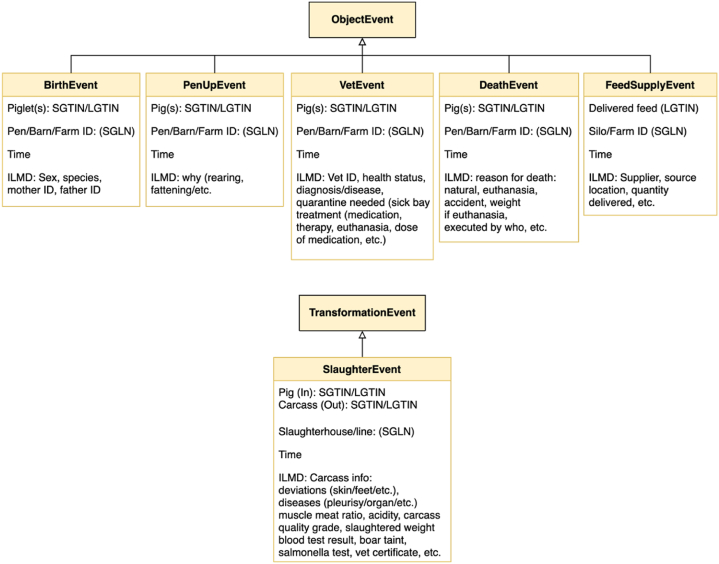
A data model for transparency events in pork supply chains.

In Fig. 14, six of the several events that can be captured across the pork supply chain are shown. Several other events such as shipping and receiving events (which occur when pigs are moved from one farm to another or to a slaughterhouse), weaning event that registers the date piglets are separated from their mother, growth event whereby animals are weighted and their growth rate is recorded, feeding and drinking events which are registered in experimental farms.

Several of the events in the framework are derived from the *ObjectEvent*. In the slaughterhouse where several transformations occur, events used in the framework are derived the *TransformationEvent*. In the model shown in Fig. 14 the most significant transformation event that occurs in the pork chain is the *SlaughterEvent*. The aggregation, transaction and associated events that occur within the pork chain are similar to those occurring in other sectors, and thus are not included in the figure. The events described below are initially modelled within the context of the IoF2020 project [57].

Animals can be identified individually or as a group depending on the level of automation practised at the farm. The *BirthEvent* is registered when piglets are born. Piglets are not tagged individually; therefore, they are identified as a group using LGTIN, which is a combination of GTIN (product type ID) and a batch identifier. Once the pigs are weaned, they can be identified either by their batch (using LGTIN) or individually (using SGTIN) in *PenUpEvent*, *VetEvent* and *DeathEvent* when transferring or inspecting them, or when they die at the farm. When feed is supplied the batch number of the feed type encoded as LGTIN is recorded together with time and the location of delivery. The timestamp of the occurrence of the events, and often together with the time the event is recorded, is identified by the datetime and the time zone.

Most events occurring at a farm (birth, relocating, vet visit or death) are associated with a specific pen, barn or farm. Places at the farm are identified using the business identification number, GLN, or a specific location at the farm (barn or pen) using SGLN. The location for feed delivery is either the farm or the specific silo, which will be identified by GLN or SGLN.

Detailed information related to the event (*why* the event is recorded) is captured as an Instance or Lot Master Data (ILMD). At birth, the gender and the generic information (mother, father, and breed) of the piglets are recorded. When relocating pigs, the reasons for relocation are recorded. When feed is supplied, the supplier's information, type and quantity of feed are recorded. Extensive ILMD data is recorded during a veterinary visit or when an animal dies at the farm.

The *SlaughterEvent* is a transformation event, therefore the ID of the input (the identity of the pig) and the IDs of the resulting products (carcass, organs, *etc.*) are used as the *what* of the event. The place and time of the event are registered in a similar fashion as other events. The slaughter event involves a large amount of ILMD, including diverse quality and safety information.

## 6 Discussion

In this paper, we presented a framework to model existing and desired future tracking and tracing systems in pork supply chains and used a case study in Vietnam to demonstrate the framework. Most pork meat samples sold in Vietnam do not meet national food quality standards, and consumers have legitimate meat quality and safety concerns [15,16]. The paper attempted to address the challenge that stakeholders in the pork supply face in modelling the existing and designing improved transparency systems by providing a consistent set of modelling approaches as a modelling framework.

The framework presents five hierarchical model types namely the domain, product flow, business control, business process and data views. These views can be used to progressively model the pork value chain in more detail and help the various stakeholders collaborate in diagnosing the issues related to transparency and designing new solutions. The models vary from the high-level domain model that can be used by all stakeholders to the more detailed business process and data models which are essential for solution providers who implement the desired transparency system.

A domain model is a simple and informative boxes-and-lines model that all actors can understand and collectively co-create in order to see how their supply chain functions or how it should operate in the future. The boxes-and-lines model is also widely used as a conceptual model. The product flow model helps to identify the interlinked value adding business processes. In this model product identification points are indicated showing whether products are identified as individual items or as a batch. The identification mechanism allows to see if product mixing or disaggregation takes place and how products are made from raw materials. Stakeholders responsible for labelling and identifying products benefit from product flow models.

Change in the supply chain management leads to changes in the control model of the supply chain. Implementation of an improved transparency system requires the scrutiny of, and when necessary changes in, the control model. For instance, better tracking of customer specific products needs changes in the customer order decoupling point in the supply chain, which in turn may necessitate data related to the business control decisions (for instance, purchase order) to be captured as part of the transparency information.

Business process and data models are essential inputs in the implementation of any information system, including transparency systems. Transparency requires collaboration and therefore adoption of global standards is essential in realizing transparency and traceability. Based on the SCOR and GS1 standards, we provided business process and data models for analysing existing and realizing new transparency systems in the pork chain. The framework provided data models that are unique to the sector. For instance, though the GS1 EPCIS events, such as the object and transformation events, were designed to be universal, the modelling of events such as the *birth*, *vet* and *slaughter* events made the models more accessible and understandable to the stakeholders in the sector and made our communication with them easier.

A key advantage of the framework is that it helps to model the architecture of a supply chain traceability system in a timely, punctual and coherent way. It combines domain-specific knowledge with knowledge from literature, generic standards, existing frameworks and reference models, and has added views that allow to model supply chain traceability systems. Table 1 summaries its main novelties compared with related solutions. As such, it enhances a shared understanding and the reuse of both cross-industry and sector-specific knowledge in the design and development of supply chain information systems in general and traceability systems in particular.

This paper builds on several previous studies [19,24,36,52,58] and has therefore benefited from a strong foundation. Nevertheless, there may sources of weaknesses that future research should address. The demonstration of the modelling approach covered only a segment of the pork chain, focussing on farming and slaughtering. More studies covering various emerging markets and the broader segment of pork supply chains (from feed up to retail and consumption) are needed to enhance the modelling framework. Furthermore, the study is based on a case study within one country. However, the need for transboundary transparency systems is essential, as the spread of the African swine fever outbreak has abundantly demonstrated. The pork value chain is global where cross-border animal trading occurs, and meat products are sold to places far away from where the animals are raised, or the meat is processed. In addition, the framework focuses on functional aspects and does not cover aspects of technical infrastructure, including privacy, permission, and security.

Another area for future research is to expand the number of views to encompass the entire software lifecycle. Currently, the views of the framework concentrate on the business part of software architecture, including data models. Models for the technical implementation such as the deployment and technical communication diagrams have not yet been included but can be adopted from existing architecture frameworks.

## 7 Conclusion

In this paper we presented a modelling framework for transparency systems in the pork sector with a specific focus on emerging markets. We described the designed framework and demonstrated how the framework can be applied through a case study. The framework presented provides a set of modelling views that address the concerns of the stakeholders in the sector. The views are adapted and extended from existing modelling views in order to adequately model and represent chain-wide transparency in supply chains.

The application of the framework is based on a case study in Vietnam. It involves three supply chain scenarios found in the Vietnamese pork sector: (1) traditional wet market supply chains that are generally open for all stakeholders to join, but uncontrolled in terms of quality and food safety, (2) integrated supply chains that are largely controlled by a central controlling company, and (3) emerging modern collaborative supply chains, that differ from integrated supply chain in the sense that each supply chain participant can decide to join. A detailed design of the collaborative supply chain is included in the present paper, since it represents a desired future scenario of open supply chains of independent members that closely work together to provide safe and traceable pork meat to consumers in emerging markets. The applicability of the framework is however not only limited to the emerging markets or to the pork sector only. As demonstrated in a previous study, the transparency system developed for the pork sector has served as a foundation for implementing a transparency system in the fruit (table olives) sector. The framework presented in this study can serve as a foundation for other food products. To achieve this, future research should provide additional validations covering diverse case studies across multiple countries in emerging markets.

## Data availability statement

The authors do not have permission to share interview transcripts. All other data is referenced in article.

## CRediT authorship contribution statement

**Ayalew Kassahun:** Writing – review & editing, Writing – original draft, Visualization, Validation, Supervision, Methodology, Investigation, Formal analysis, Data curation, Conceptualization. **Cor Verdouw:** Writing – review & editing, Writing – original draft, Visualization, Validation, Methodology, Investigation, Formal analysis, Conceptualization. **Jeroen Roomer:** Writing – review & editing, Validation, Supervision, Software, Resources, Project administration, Investigation, Data curation, Conceptualization.

## Declaration of competing interest

The authors declare the following financial interests/personal relationships which may be considered as potential competing interests:**Jeroen Roomer** is the Chief Information Officer (CIO) of the case study company, De Heus Animal Nutrition B.V., Ede, The Netherlands (https://www.de-heus.nl/). He is also the responsible person who oversaw the development of the Farm Management Information Systems (FMIS) for farmers and the transparency system for the independent collaborative supply chain orchestrator, used in the case study.**Cor Verdouw** is also an Innovation Manager at Mprise Agriware B.V., Veenendaal, The Netherlands (https://www.mprise.nl/en/), besides his role as a Senior Scientist at Wageningen University.However, the manuscript is in no way aimed at promoting the case study company, or is related to any financial aspect of both companies. At most care has been given to make the case study as anonymously as possible while keeping the relevant details.

## Figures and Tables

**Table A1:** Main modelling elements and relations of domain model

Symbol	Name	Description
	Producer	Supply chain actor that transforms inputs into new products (raw materials, half fabricates or end products)
	Farmer	Producer that transforms farm inputs into agricultural products such as animals or crops.
	Intermediary	Supply chain actor that provides services but that do not transform products, including logistic service providers (transportation, storage facilities, etc.) and traders
	Retailer	Supply chain actor that distributes and sells end products to consumers
	Coordination	Coordinated part of the supply chain, in which the actors collaborate and the processes are aligned
	Product flow	Primary physical stream within the supply chain from raw materials into products for end customers

**Table A2:** Main modelling elements and relations of a product flow model

Symbol	Name	Description
	System boundary	Boundary of the supply chain in scope
	External stakeholder	Actor out of scope, *e.g.* input suppliers and end customers
	Supply chain actor	Supply chain actor within scope
	Value-adding process	Primary business process that add value to the end customer, based on SCOR level 1
	Product flow	Material, half fabricates or (by-) products
	Storage	Strategic inventory point of material, half fabricates or (by-) products
	Traceability units	Levels of product granularity for traceability, including Shipping Units (SU), Logistic Units (LU), Trade Units (TU) and Consumer Units (CU)

**Table A3:** Main modelling elements and relations of a thread diagram (in addition to the product flow model)

Symbol	Name	Description
	Business control process	Business process that is needed to control a value-adding process, based on SCOR level 2
	Order trigger	External event that kicks off a (cycle of) business control processes based on an order between external stakeholders and/or internal supply chain actors
	Time trigger	Internal event that kicks off a (cycle of) business control processes based on own demand forecast of a supply chain actor
	CODP	Customer Order Decoupling Point, strategic inventory that decouples order-driven and forecast-driven business control processes
	Traceability data store	An enterprise repository to register, manage and retrieve traceability data
	Control sequence flow	Sequence flow that connects a cycle of order-driven business processes. Such a cycle starts with a trigger (order or time) and ends with the execution of value-adding business process or with starting a new order trigger for another supply chain actor.
	Traceability data flow	Data flow that connects data stores with business control processes or other data stores. The direction of the flow indicates whether data is input (register/update) or output (retrieve) of the data store.

**Table A4:** Main modelling elements and relations of business process model, adopted from BPMN 2.0

Symbol	Name	Description
	Start event	Event that triggers the process, either by a message or by a cycle/schedule (timer) or unspecified.
	Intermediate event	Internal event that triggers a task within the process, either by a message, by a cycle/schedule (timer) or unspecified.
	End event	Final outcome that stops the process.
	Task	Activity which has to be executed by a human performer or a resource like a machine, define on SCOR level 3 or beyond
	Information task	Task to register, process, share or retrieve information, used to represent a user action in a (software) system of an fully automated information action.
	Production task	Manual task used to represent a (usually physical) production action
	Gateway	Decision points that determine what path is taken through a process, by either exclusive (X: choice), parallel (+) or unspecified controls of sequence flows.
	Data store	A data repository from/to which an activity can read from or write to data that must remain to exist after the termination of the process.
	Data object	Information elements like documents or files that are used as an input or an output of an activity within the process
	Message	Information elements like documents or files that are exchanged between pools and thus define communication between supply chain actors or systems
	Sequence flow	Connectors which are mandatory and show the order in which activities are performed
	Association	Connectors which are used to connect data objects to process elements
	Message flow	Connectors which are used to show communication between pools
	Pool	A pool represents an organization (company, business unit) or a system that is responsible for a process
	Lane	A sub-partition within a pool that specifies the roles within an organization or modules within a system

**Table A5:** Main modelling elements and relations of a data model

Symbol	Name	Description
	Class	An object that represents important data. Each class consists of a header and a list of attributes, including class type, a key attribute (unique identifier), and other properties.
	Inheritance	Class is a specialized form of the other class.
	Aggregation	Class is a collection or container of the other class.
